# Peer Learning and Operationalizing During COVID-19 Pandemic and Beyond

**DOI:** 10.7759/cureus.16568

**Published:** 2021-07-22

**Authors:** Mayur Virarkar, Ajaykumar C Morani, Priya Bhosale, Nicolaus A Wagner-Bartak, Brett W Carter, Elizabeth Lano

**Affiliations:** 1 Radiology, The University of Texas Health Science Center at Houston, Houston, USA; 2 Abdominal Imaging, The University of Texas MD Anderson Cancer Center, Houston, USA; 3 Thoracic Imaging, The University of Texas MD Anderson Cancer Center, Houston, USA

**Keywords:** peer learning, covid-19, acr, scores, virtual conference

## Abstract

The main objective of the article is to describe the changes in managing the peer learning system in the Department of Abdominal Imaging at our institution during the pandemic and its restrictions. The pandemic poses diverse challenges to academic institutions across the country including radiology education and peer learning. The health sector in some areas of the country has been stretched by the number of coronavirus disease 2019 (COVID-19) patients. In March 2020, our institution cancelled all in-person conferences as per guidelines from the Center of Disease Control and Prevention to mitigate the spread of COVID-19 and the conferences were shifted to virtual platforms. Our recent peer learning approach allowed us to practice appropriate social distancing while following the institutional and national guidelines with minimal disruption. Other institutions that are facing similar challenges can adopt or modify our framework of a successful and efficient virtual peer learning process.

## Introduction

The World Health Organization officially declared a global pandemic on March 11, 2020 due to rapidly increasing coronavirus disease 2019 (COVID-19) cases worldwide [1]. There has been a tremendous increase in viral spread in the United States with close to 34,000,000 cases and over 600,000 deaths by June 2021 [2]. The healthcare sector has been pressured and pushed to critical levels in some areas due to the number of COVID-19 patients. These developments have had repercussions on academic and non-academic institutions. In addition, the pandemic poses extraordinary challenges to radiology services, educational and quality improvement activities, including peer learning [3]. The development and implementation of COVID-19 vaccines is envisioned as a key intervention to significantly reduce the threat of the virus.

In 2002, RADPEER™ was introduced by the American College of Radiology (ACR) to provide quality assessment and improvement through routine peer review of radiologists [4]. It includes a score-based retrospective evaluation of previously interpreted imaging studies. Although it is a widely used system and a requirement for credentialing with the Joint Commission (TJC) [5], there has been debate regarding its yield in identifying meaningful learning opportunities [6]. In September 2015, the Institute of Medicine published a report titled “Improving Diagnosis in Health Care,” which highlighted the importance of learning from diagnostic error, declaring it a moral, professional, and public health imperative [7]. It also shifted the focus on mistakes from an individual in traditional peer review processes to a learning medium for the group. In 2016, Larson and colleagues offered an alternative approach to peer review: peer feedback, learning, and improvement (or more succinctly, “peer learning”) with the principles promoted by the Institute of Medicine [8]. Since its introduction, the peer learning system has demonstrated increased learning opportunities and promoted a culture of learning and improvement [9-12].

In the article, we layout the peer learning system in our institution, and the major difficulties faced during the pandemic and their countermeasures.

## Materials and methods

Background

In our Department of Abdominal Imaging, the primary goal of peer learning system is to improve patient care through studying of learning opportunities. This process reinforces a "just culture" of safety, quality, education, and accountability, and is intended to be educational, non-punitive, and positive [13]. The peer learning system meets the requirements for physician peer review stipulated by accrediting organizations and professional societies such as the Centers for Medicare and Medicaid Services [14], TJC [5], the ACR [4,15], and the American Board of Radiology [16].

The process

**Figure 1:**
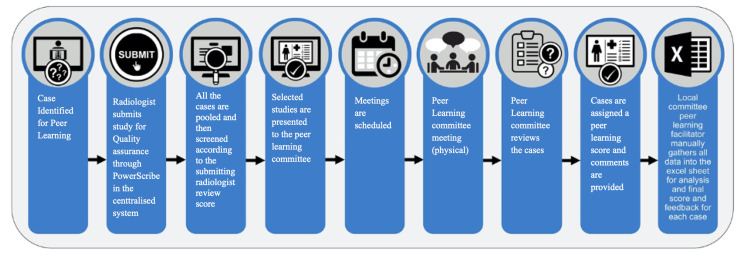
Baseline peer learning system at our institution

The peer learning system is a two-step process (Figure 1) [17-19]. The first step consists of the mandatory and voluntary submission of cases by faculty members or trainees. The second step involves the formal evaluation of submitted cases with learning opportunities by a committee composed of department faculty members. The peer learning committee must meet at least quarterly to review the cases that have been submitted for evaluation. In our department, the meetings are held monthly to review the submitted cases.

Submission of cases

**Figure 2:**
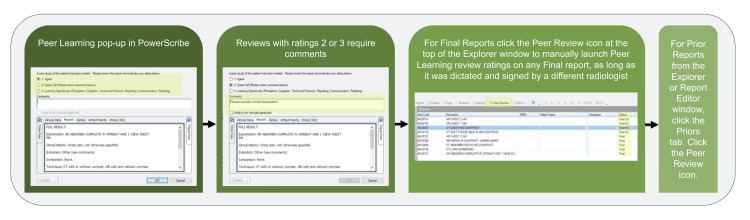
Submission of cases through PowerScribe

Most cases are submitted by the radiologists interpreting imaging studies using the voice recognition system employed in the department (PowerScribe, Nuance) (Figure 2). The radiologists are prompted to submit a randomly selected prior case for every 20th case that is dictated and signed. The ACR does not require a minimum number of reviews per physician. The group may optionally elect to choose a target number of peer submissions as part of your internal quality assurance program. The goal for the department decided for a minimum 5% of cases to be submitted and reviewed. The radiologists interpreting a study may also voluntarily electronically submit any prior case for peer learning through PowerScribe. Additional voluntary cases submitted to peer learning system include learning opportunities derived from discussions at multidisciplinary tumor boards and other conferences, consultations with referring providers, and discussions with other radiologists.

Scoring system

The peer learning system consists of the following scores: 1) agree; 2) great call (positive feedback); and 3) learning opportunity, with specific subdivisions based on the type of learning opportunity (perception, cognition, technical/protocol, reporting, communication, radiologist recommendation, and other process related) [15,18] (Table 1).

**Table 1:** Rankings associated with peer learning system

Rating Definition	Score	Comment Required?
Agree	1	No
Great Call	2	Yes
Learning Opportunity: Perception Cognition Technical/Protocol Reporting Communication Radiologist Recommendation Other Process Related	3	Yes

Review of cases

A list of cases submitted for peer learning are forwarded to the peer learning faculty lead in the department by the Patient Safety Coordinator in Diagnostic Imaging. Each conference is facilitated by this local leader and consists of members of the abdominal imaging department. The meetings are open to all department faculty members. For each case, the categorization and comments submitted by the original reviewer are evaluated. The physicians participating in the process select a score independently, and a final score is assigned to the case based on the category most frequently selected by the group. The cases with final scores and comments are submitted to the Patient Safety Coordinator in Diagnostic Imaging.

Radiologist notification of results

Faculty members and member submitting the peer review are notified of the final results of cases that they have interpreted and subsequently reviewed by the peer learning committee. In 2019, a database of cases reviewed and assigned a final peer learning score by each committee was created and is continuously updated with detailed information regarding cases that are reviewed. This database can be accessed by each faculty member so they can see their individual peer learning reports. The radiologist can also opt to receive an automated monthly email update.

## Results

Peer learning in COVID era

In March 2020, our institution cancelled all in-person conferences as per guidelines from the Center of Disease Control and Prevention to mitigate the spread of COVID-19. These conferences were shifted to virtual platforms such as WebEx (Cisco, California, USA) or Zoom (Zoom, California, USA) [3]. Furthermore, when feasible, our radiologists started reading remotely from home to adhere to social distancing guidelines. This posed new challenges for continuing radiology educational activities in our institution including peer learning. In response, the peer learning activities were modified to comply with the institutional guidelines and the evolving circumstances.

**Figure 3:**
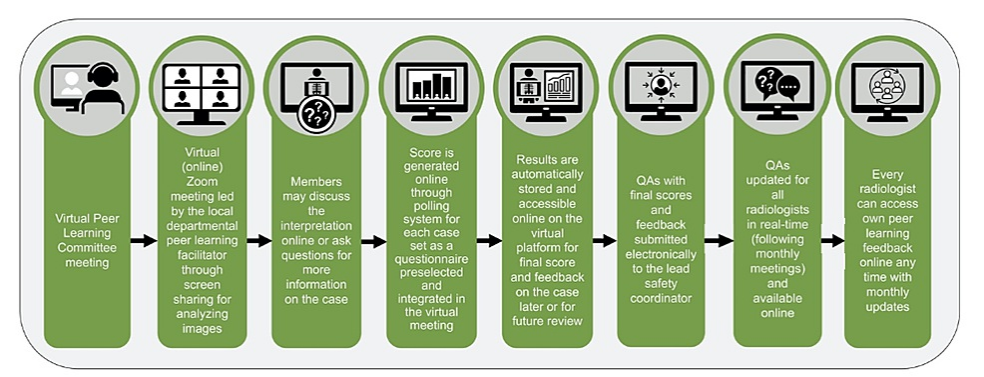
Adapted new peer learning system at our institution

The Department of Abdominal Imaging switched the in-person peer learning meetings to virtual conferences (Figure 3).

**Figure 4:**
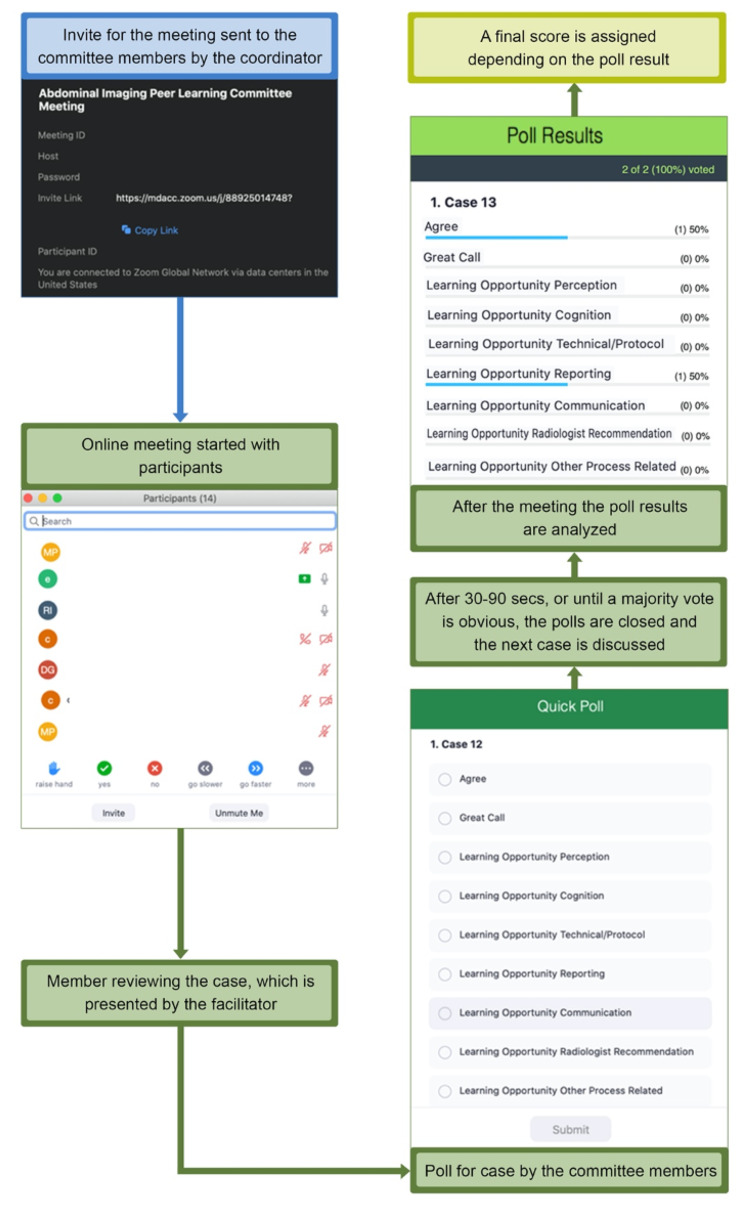
Peer review virtual meeting at our institution

The radiologist assigned as the peer learning faculty lead in the department retained the responsibility of the meeting. The meeting was held virtually once a month for 1 hour. The lead organized and scheduled the virtual meeting through their institutional account for the virtual platform and sent invitations to the committee members (Figure 4).

Through the simple pre-selected settings of the virtual meetings, and for security and protected health information purposes, only the registered and authenticated members were allowed to enter these virtual meetings.

## Discussion

The virtual-meeting platform settings enabled the automatic collection of the number and names of attendees for continued medical education (CME) and risk management education (RME) credits reporting, the time of attendance of each attendee, individual score, as well as the final poll results on the pear learning score for each case reviewed. During the meeting, the lead will review the cases by sharing their picture archiving and communication systems screen and also answer any query pertaining to the case and imaging findings. After each case, the committee members score the case via an online poll. After 30-90 seconds or at a majority vote, the poll is closed for responses and the next case is discussed. In a single meeting, 20-25 cases are discussed, and poll results are obtained for each case. After the meeting, the lead analyzes the responses, and a final category is assigned to the case based on the poll results. The online poll negated the need of the hardcopy of the questionnaires with responses of the committee members. In addition to expedited tabulation of poll results, the electronic data can be automatically stored for evaluation later or exported into a computerized database.

We moved from our prior in-person peer learning process to a completely virtual peer learning system given the restrictions during the COVID-19 pandemic. This allowed us to practice social distancing by avoiding in-person meetings but does not inhibit our ability to review cases and collect feedback and final scores on each case. The meetings are easy to set up and offer the needed security to protect patient data. The electronic polls further save personnel time by avoiding manual sorting and analyzing of each attendees’ response on every case. Electronic attendee data help streamline reporting for CME and RME credits. Further, virtual meetings have proven convenient and favored by attendees who can log in from anywhere into the password-protected meeting at their convenience, which has resulted in a higher attendance rate.

Limitations

There are also a few barriers for the all-virtual peer learning process. The first and foremost is learning and adjusting to new technology. But, as this process is relatively simple to set up and use, it did not require a significant investment of time or resources to roll out. Secondly, the initial few meetings did show a slightly reduced efficiency with approximately 5% fewer cases reviewed per meeting, until all attendees familiarized themselves with the process and technology.

## Conclusions

In these difficult times, it remains essential to maintain radiology education and quality activities, including peer learning. This article presents some countermeasures adopted by our institution to continue peer learning. We believe that our approach allows us to practice social distancing and follow institutional and national guidelines with minimal disruptions to our quality and educational activities. Other institutions that are facing similar challenges can use our framework of a successful and efficient virtual peer learning process for increasing learning opportunities and continuing a culture of safety and improvement.
